# Bis(μ-3-carb­oxy-2-hy­droxy­propane-1,2-dicarboxyl­ato)bis(diaquazinc)–1,2-bis­(pyridin-4-yl)ethene–water (1/1/2)

**DOI:** 10.1107/S1600536812039761

**Published:** 2012-09-26

**Authors:** In Hong Hwang, Pan-Gi Kim, Cheal Kim, Youngmee Kim

**Affiliations:** a Department of Fine Chemistry, Seoul National University of Science and Technology, Seoul 139-743, Republic of Korea; b Department of Forest and Environment Resources, Kyungpook National University, Sangju 742-711, Republic of Korea; c Department of Chemistry and Nano Science, Ewha Women’s University, Seoul 120-750, Republic of Korea

## Abstract

The asymmetric unit of the title compound, [Zn_2_(C_6_H_6_O_7_)_2_(H_2_O)_4_]·C_12_H_10_N_2_·2H_2_O, comprises half of a centrosymmetric complex dimer, half of a 1,2-bis­(pyridin-4-yl)ethene mol­ecule, which lies across an inversion centre, and one lattice water mol­ecule. Carboxyl­ate groups of two dianionic citrate ligands bridge two Zn^II^ ions to give the cyclic dimer, with each Zn^II^ ion coordinated by four O atoms from the chelating citrate ligand (one hy­droxy and three carboxyl­ate, with one bridging) and two water O atoms, forming a distorted octa­hedral environment [Zn—O = 2.040 (3)–2.244 (3) Å]. In the crystal, O—H⋯O and O—H⋯N hydrogen bonds involving hy­droxy groups and both coordinating and lattice water mol­ecules link the dimers to give a three-dimensional framework structure.

## Related literature

For inter­actions of metal ions with biologically active mol­ecules, see: Daniele *et al.* (2008 ▶); Parkin (2004 ▶); Tshuva & Lippard (2004 ▶); Stoumpos *et al.* (2009 ▶). For a manganese citrate complex, see: Hwang *et al.* (2012 ▶). For related complexes, see: Shin *et al.* (2009 ▶); Yu *et al.* (2009 ▶); Kim *et al.* (2011 ▶).
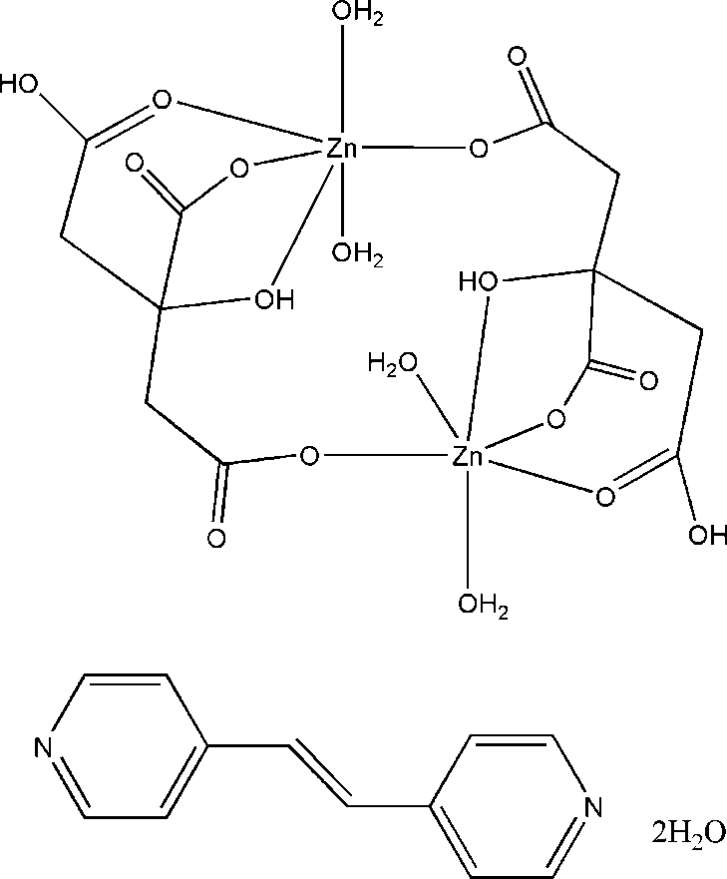


## Experimental

### 

#### Crystal data

[Zn_2_(C_6_H_6_O_7_)_2_(H_2_O)_4_]·C_12_H_10_N_2_·2H_2_O
*M* *_r_* = 801.31
Triclinic, 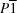

*a* = 9.4360 (19) Å
*b* = 9.4540 (19) Å
*c* = 10.098 (2) Å
α = 66.87 (3)°
β = 70.19 (3)°
γ = 75.91 (3)°
*V* = 773.0 (3) Å^3^
*Z* = 1
Mo *K*α radiation
μ = 1.64 mm^−1^
*T* = 170 K
0.30 × 0.10 × 0.10 mm

#### Data collection

Bruker SMART CCD diffractometer
Absorption correction: multi-scan (*SADABS*; Bruker, 1997 ▶) *T*_min_ = 0.638, *T*_max_ = 0.853
4216 measured reflections
2924 independent reflections
2382 reflections with *I* > 2σ(*I*)
*R*_int_ = 0.034

#### Refinement

*R*[*F* ^2^ > 2σ(*F* ^2^)] = 0.051
*wR*(*F* ^2^) = 0.140
*S* = 1.09
2924 reflections
239 parameters
7 restraints
H atoms treated by a mixture of independent and constrained refinement
Δρ_max_ = 0.77 e Å^−3^
Δρ_min_ = −1.36 e Å^−3^

### 

Data collection: *SMART* (Bruker, 1997 ▶); cell refinement: *SAINT* (Bruker, 1997 ▶); data reduction: *SAINT*; program(s) used to solve structure: *SHELXS97* (Sheldrick, 2008 ▶); program(s) used to refine structure: *SHELXL97* (Sheldrick, 2008 ▶); molecular graphics: *SHELXTL* (Sheldrick, 2008 ▶); software used to prepare material for publication: *SHELXTL* .

## Supplementary Material

Crystal structure: contains datablock(s) I, global. DOI: 10.1107/S1600536812039761/zs2234sup1.cif

Structure factors: contains datablock(s) I. DOI: 10.1107/S1600536812039761/zs2234Isup2.hkl

Additional supplementary materials:  crystallographic information; 3D view; checkCIF report

## Figures and Tables

**Table 1:** Hydrogen-bond geometry (Å, °)

*D*—H⋯*A*	*D*—H	H⋯*A*	*D*⋯*A*	*D*—H⋯*A*
O1—H1*O*⋯O6	0.93 (1)	1.80 (2)	2.626 (4)	146 (4)
O5—H5⋯N11^i^	0.84	1.81	2.633 (5)	168
O8—H8*B*⋯O5^i^	0.86 (1)	1.88 (1)	2.733 (5)	173 (5)
O8—H8*A*⋯O7^ii^	0.86 (1)	2.04 (2)	2.866 (4)	161 (5)
O9—H9*B*⋯O1*W* ^ii^	0.86 (1)	1.87 (1)	2.725 (5)	179 (5)
O9—H9*A*⋯O3^iii^	0.86 (1)	2.57 (4)	3.145 (5)	125 (4)
O9—H9*A*⋯O2^iii^	0.86 (1)	2.01 (1)	2.860 (5)	168 (5)
O1*W*—H1*WA*⋯O3	0.96 (1)	1.88 (1)	2.838 (5)	172 (5)
O1*W*—H1*WB*⋯O7^iv^	0.96 (1)	2.04 (3)	2.880 (5)	145 (4)

Symmetry codes: (i) 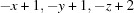; (ii) 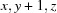; (iii) 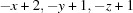; (iv) 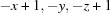.

**Fig. 1.**
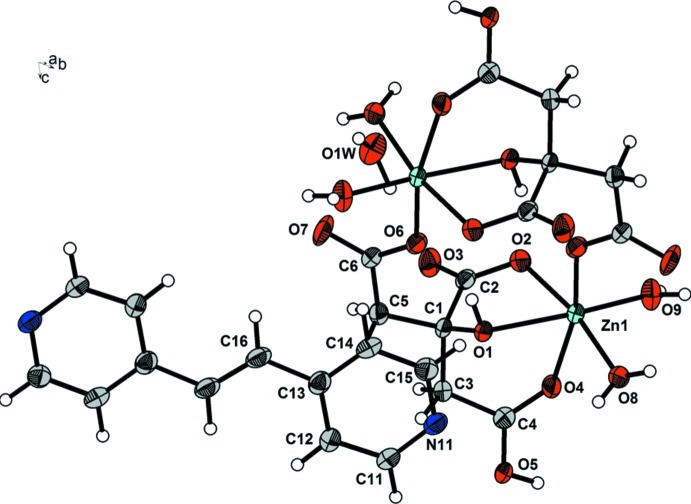
The structure of the title compound showing the atom numbering scheme. Displacement ellipsoids are shown at the 50% probability level. The labelled atoms are related with unlabelled atoms by symmetry code: -x + 1, -y + 1, -z + 1 for the diaquabis(citrato)dizinc fragment and -x, -y, -z + 2 for the 1,2-bis(pyridin-4-yl)ethene molecule.

## supplementary crystallographic information

### Comment

Citric acid has often been used as a model ligand to examine the interaction between transition metal ions with biologically active molecules (Daniele *et al.*, 2008; Parkin, 2004; Tshuva & Lippard, 2004; Stoumpos *et al.*, 2009). Recently, our group has also reported a novel compound from the reaction of manganese(II) nitrate as a building block and citric acid as a ligand (Hwang *et al.*, 2012). In order to study the effects of secondary metal ions on the interaction between transition metal ions and citric acid (Shin *et al.*, 2009; Yu *et al.*, 2009; Kim *et al.*, 2011), we have employed zinc as a metal ion source. We report here the structure of [Zn_2_(H_2_O)_4_(C_6_H_8_O_7_)_2_].C_12_H_10_N_2_.2H_2_O.

In the structure of the title compound (Fig. 1) the asymmetric unit contains half of a centrosymmetric complex dimer, half of a 1,2-bis(pyridin-4-yl)ethene molecule which lies across an inversion centre and one water molecule. Carboxylate groups of two dianionic citrate ligands bridge two Zn^II^ ions giving the cyclic dimer, with each ZnO_6_centre coordinated by four O atoms from the ligand (one hydroxyl and three carboxyl) and two water O atoms, forming a distorted octahedral environment [Zn—O, 2.040 (3)–2.244 (3) Å]. In the crystal, O—H—O and O—H···N hydrogen bonds involving hydroxyl groups and both coordinated and solvent water molecules (Table 1) link the dimers giving a three-dimensional framework structure. The crystal structure is further stabilized by weak intermolecular π–π interactions involving the 1,2-bis(pyridin-4-yl)ethene molecule [centroid = C11–C15/N11; ring centroid separation = 3.97 (7) Å; symmetry code: -*x*, -*y* + 1, -*z* + 2].

### Experimental

Citric acid (19.4 mg, 0.1 mmol) and Zn(NO_3_)_2_.6H_2_O (30.4 mg, 0.1 mmol) were dissolved in 4 ml of H_2_O and carefully layered by 4 ml of an acetonitrile solution of 1,2-bis(4-pyridyl)ethylene (37.6 mg, 0.2 mmol). Suitable crystals of the title compound were obtained in a month.

### Refinement

H atoms bonded to carbon were placed in calculated positions with C—H = 0.95 Å (aromatic C) and 0.99 Å (methylene C) and were included in the refinement in a riding-motion approximation with *U*_iso_(H) = 1.2*U*_eq_(C). The H atom bonded to the carboxylate O was placed in a calculated position with O—H = 0.84 Å and was also included in the refinement in the riding-motion approximation with *U*_iso_(H) = 1.5*U*eq(O). The position of hydroxyl H atom was refined with O—H = 0.93 Å and *U*_iso_(H) = 1.5*U*_eq_(O). The positions of O—H atoms of the coordinated water ligands were refined with O—H = 0.86 Å and *U*_iso_(H) = 1.2*U*_eq_(N). The positions of O—H atoms of the free water molecule were refined with O—H = 0.96 Å and *U*_iso_(H) =1.2*U*_eq_(O).

### Crystal data

**Table:**

[Zn_2_(C_6_H_6_O_7_)_2_(H_2_O)_4_]·C_12_H_10_N_2_·2H_2_O	*Z* = 1
*M**_r_* = 801.31	*F*(000) = 412
Triclinic, *P*1	*D*_x_ = 1.721 Mg m^−^^3^
Hall symbol: -P 1	Mo *K*α radiation, λ = 0.71073 Å
*a* = 9.4360 (19) Å	Cell parameters from 11909 reflections
*b* = 9.4540 (19) Å	θ = 2.7–27.6°
*c* = 10.098 (2) Å	µ = 1.64 mm^−^^1^
α = 66.87 (3)°	*T* = 170 K
β = 70.19 (3)°	Block, colourless
γ = 75.91 (3)°	0.30 × 0.10 × 0.10 mm
*V* = 773.0 (3) Å^3^	

### Data collection

**Table:**

Bruker SMART CCD diffractometer	2924 independent reflections
Radiation source: fine-focus sealed tube	2382 reflections with *I* > 2σ(*I*)
Graphite monochromator	*R*_int_ = 0.034
φ and ω scans	θ_max_ = 26.0°, θ_min_ = 2.3°
Absorption correction: multi-scan (*SADABS*; Bruker, 1997)	*h* = −11→6
*T*_min_ = 0.638, *T*_max_ = 0.853	*k* = −11→11
4216 measured reflections	*l* = −12→12

### Refinement

**Table:**

Refinement on *F*^2^	Primary atom site location: structure-invariant direct methods
Least-squares matrix: full	Secondary atom site location: difference Fourier map
*R*[*F*^2^ > 2σ(*F*^2^)] = 0.051	Hydrogen site location: inferred from neighbouring sites
*wR*(*F*^2^) = 0.140	H atoms treated by a mixture of independent and constrained refinement
*S* = 1.09	*w* = 1/[σ^2^(*F*_o_^2^) + (0.0822*P*)^2^] where *P* = (*F*_o_^2^ + 2*F*_c_^2^)/3
2924 reflections	(Δ/σ)_max_ < 0.001
239 parameters	Δρ_max_ = 0.77 e Å^−^^3^
7 restraints	Δρ_min_ = −1.36 e Å^−^^3^

### Special details

**Table:**

Geometry. All e.s.d.'s (except the e.s.d. in the dihedral angle between two l.s. planes) are estimated using the full covariance matrix. The cell e.s.d.'s are taken into account individually in the estimation of e.s.d.'s in distances, angles and torsion angles; correlations between e.s.d.'s in cell parameters are only used when they are defined by crystal symmetry. An approximate (isotropic) treatment of cell e.s.d.'s is used for estimating e.s.d.'s involving l.s. planes.
Refinement. Refinement of *F*^2^ against ALL reflections. The weighted *R*-factor *wR* and goodness of fit *S* are based on *F*^2^, conventional *R*-factors *R* are based on *F*, with *F* set to zero for negative *F*^2^. The threshold expression of *F*^2^ > σ(*F*^2^) is used only for calculating *R*-factors(gt) *etc*. and is not relevant to the choice of reflections for refinement. *R*-factors based on *F*^2^ are statistically about twice as large as those based on *F*, and *R*-factors based on ALL data will be even larger.

### Fractional atomic coordinates and isotropic or equivalent isotropic displacement parameters (Å^2^)

**Table:**

	*x*	*y*	*z*	*U*_iso_*/*U*_eq_	
Zn1	0.68767 (6)	0.59099 (5)	0.60957 (5)	0.02122 (19)	
O1	0.5132 (3)	0.4257 (3)	0.7203 (3)	0.0209 (6)	
H1O	0.466 (4)	0.431 (5)	0.651 (4)	0.031*	
O2	0.8003 (4)	0.3981 (3)	0.5435 (3)	0.0260 (7)	
O3	0.8228 (4)	0.1376 (3)	0.6320 (4)	0.0341 (8)	
O4	0.7490 (4)	0.4656 (3)	0.8125 (3)	0.0282 (7)	
O5	0.6894 (4)	0.3353 (3)	1.0577 (3)	0.0310 (7)	
H5	0.7402	0.3981	1.0558	0.046*	
O6	0.4181 (4)	0.3256 (3)	0.5605 (3)	0.0262 (7)	
O7	0.4259 (5)	0.0698 (3)	0.6153 (4)	0.0427 (9)	
O8	0.5622 (4)	0.7538 (3)	0.7112 (3)	0.0287 (7)	
H8A	0.542 (6)	0.8518 (11)	0.668 (5)	0.034*	
H8B	0.480 (3)	0.733 (5)	0.782 (4)	0.034*	
O9	0.8800 (4)	0.7008 (3)	0.5133 (4)	0.0333 (7)	
H9A	0.9753 (13)	0.675 (6)	0.507 (6)	0.040*	
H9B	0.866 (6)	0.8005 (4)	0.482 (5)	0.040*	
C1	0.5996 (5)	0.2711 (4)	0.7563 (4)	0.0192 (8)	
C2	0.7522 (5)	0.2671 (4)	0.6340 (4)	0.0222 (9)	
C3	0.6235 (5)	0.2336 (4)	0.9105 (4)	0.0249 (9)	
H3A	0.5242	0.2187	0.9867	0.030*	
H3B	0.6906	0.1340	0.9338	0.030*	
C4	0.6919 (5)	0.3555 (4)	0.9255 (4)	0.0238 (9)	
C5	0.5058 (5)	0.1490 (4)	0.7724 (4)	0.0235 (9)	
H5A	0.5694	0.0469	0.7911	0.028*	
H5B	0.4176	0.1417	0.8617	0.028*	
C6	0.4474 (5)	0.1808 (4)	0.6386 (4)	0.0228 (9)	
N11	0.1862 (4)	0.4370 (4)	0.9552 (4)	0.0282 (8)	
C11	0.1610 (6)	0.3080 (5)	1.0802 (5)	0.0300 (10)	
H11	0.1826	0.3029	1.1674	0.036*	
C12	0.1051 (5)	0.1855 (5)	1.0827 (5)	0.0290 (10)	
H12	0.0875	0.0967	1.1705	0.035*	
C13	0.0741 (5)	0.1939 (5)	0.9522 (5)	0.0271 (9)	
C14	0.0990 (5)	0.3284 (5)	0.8255 (5)	0.0280 (10)	
H14	0.0771	0.3372	0.7372	0.034*	
C15	0.1555 (5)	0.4480 (5)	0.8300 (5)	0.0285 (10)	
H15	0.1729	0.5388	0.7441	0.034*	
C16	0.0212 (5)	0.0643 (5)	0.9421 (4)	0.0297 (10)	
H16	0.0168	0.0727	0.8467	0.036*	
O1W	0.8324 (4)	0.0166 (4)	0.4122 (4)	0.0430 (9)	
H1WA	0.819 (6)	0.057 (6)	0.490 (4)	0.052*	
H1WB	0.736 (3)	0.034 (6)	0.391 (6)	0.052*	

### Atomic displacement parameters (Å^2^)

**Table:**

	*U*^11^	*U*^22^	*U*^33^	*U*^12^	*U*^13^	*U*^23^
Zn1	0.0271 (3)	0.0171 (3)	0.0208 (3)	−0.00588 (19)	−0.0053 (2)	−0.00706 (19)
O1	0.0261 (16)	0.0154 (12)	0.0223 (14)	−0.0028 (12)	−0.0067 (13)	−0.0072 (11)
O2	0.0314 (17)	0.0210 (14)	0.0240 (14)	−0.0041 (13)	−0.0066 (13)	−0.0064 (12)
O3	0.0333 (19)	0.0196 (14)	0.0436 (18)	0.0001 (13)	−0.0025 (15)	−0.0135 (13)
O4	0.0400 (19)	0.0243 (15)	0.0248 (15)	−0.0135 (14)	−0.0144 (14)	−0.0028 (12)
O5	0.047 (2)	0.0309 (15)	0.0236 (14)	−0.0232 (15)	−0.0075 (15)	−0.0089 (13)
O6	0.0399 (19)	0.0193 (13)	0.0234 (14)	−0.0032 (13)	−0.0146 (14)	−0.0066 (11)
O7	0.076 (3)	0.0190 (15)	0.0502 (19)	−0.0098 (16)	−0.037 (2)	−0.0102 (14)
O8	0.040 (2)	0.0188 (14)	0.0242 (15)	−0.0045 (14)	−0.0040 (14)	−0.0081 (12)
O9	0.0251 (17)	0.0230 (15)	0.0490 (19)	−0.0057 (14)	−0.0071 (16)	−0.0103 (14)
C1	0.025 (2)	0.0144 (17)	0.0204 (19)	−0.0048 (16)	−0.0068 (17)	−0.0062 (15)
C2	0.025 (2)	0.0218 (19)	0.023 (2)	−0.0038 (18)	−0.0084 (18)	−0.0098 (16)
C3	0.032 (3)	0.0210 (19)	0.022 (2)	−0.0100 (18)	−0.0042 (19)	−0.0062 (16)
C4	0.024 (2)	0.024 (2)	0.025 (2)	−0.0020 (18)	−0.0075 (18)	−0.0095 (17)
C5	0.028 (2)	0.0186 (18)	0.024 (2)	−0.0050 (17)	−0.0079 (18)	−0.0052 (16)
C6	0.027 (2)	0.0221 (19)	0.0231 (19)	−0.0049 (17)	−0.0074 (18)	−0.0098 (16)
N11	0.031 (2)	0.0290 (18)	0.0293 (18)	−0.0035 (17)	−0.0072 (17)	−0.0162 (16)
C11	0.039 (3)	0.029 (2)	0.028 (2)	−0.006 (2)	−0.009 (2)	−0.0143 (18)
C12	0.036 (3)	0.025 (2)	0.029 (2)	−0.0079 (19)	−0.010 (2)	−0.0094 (18)
C13	0.026 (2)	0.026 (2)	0.031 (2)	−0.0023 (19)	−0.0034 (19)	−0.0156 (18)
C14	0.031 (3)	0.031 (2)	0.028 (2)	−0.0002 (19)	−0.011 (2)	−0.0169 (18)
C15	0.028 (3)	0.030 (2)	0.028 (2)	−0.0052 (19)	−0.0042 (19)	−0.0118 (18)
C16	0.037 (3)	0.029 (2)	0.027 (2)	0.000 (2)	−0.009 (2)	−0.0172 (18)
O1W	0.042 (2)	0.0371 (18)	0.057 (2)	−0.0027 (17)	−0.0159 (19)	−0.0224 (17)

### Geometric parameters (Å, º)

**Table:**

Zn1—O9	2.060 (3)	C1—C2	1.551 (6)
Zn1—O6^i^	2.069 (3)	C3—C4	1.527 (5)
Zn1—O8	2.086 (3)	C3—H3A	0.9900
Zn1—O2	2.105 (3)	C3—H3B	0.9900
Zn1—O4	2.122 (3)	C5—C6	1.525 (5)
Zn1—O1	2.244 (3)	C5—H5A	0.9900
O1—C1	1.461 (4)	C5—H5B	0.9900
O1—H1O	0.930 (2)	N11—C15	1.351 (5)
O2—C2	1.296 (5)	N11—C11	1.366 (5)
O3—C2	1.248 (5)	C11—C12	1.374 (6)
O4—C4	1.264 (5)	C11—H11	0.9500
O5—C4	1.265 (5)	C12—C13	1.412 (6)
O5—H5	0.8400	C12—H12	0.9500
O6—C6	1.299 (5)	C13—C14	1.404 (6)
O6—Zn1^i^	2.069 (3)	C13—C16	1.481 (6)
O7—C6	1.236 (5)	C14—C15	1.384 (6)
O8—H8A	0.860 (2)	C14—H14	0.9500
O8—H8B	0.860 (2)	C15—H15	0.9500
O9—H9A	0.860 (2)	C16—C16^ii^	1.345 (8)
O9—H9B	0.860 (2)	C16—H16	0.9500
C1—C3	1.539 (5)	O1W—H1WA	0.960 (2)
C1—C5	1.547 (5)	O1W—H1WB	0.960 (2)
			
O9—Zn1—O6^i^	102.94 (13)	C4—C3—H3A	108.4
O9—Zn1—O8	94.22 (13)	C1—C3—H3A	108.4
O6^i^—Zn1—O8	94.62 (12)	C4—C3—H3B	108.4
O9—Zn1—O2	92.48 (13)	C1—C3—H3B	108.4
O6^i^—Zn1—O2	90.97 (11)	H3A—C3—H3B	107.4
O8—Zn1—O2	170.11 (11)	O4—C4—O5	123.4 (4)
O9—Zn1—O4	92.53 (13)	O4—C4—C3	121.6 (3)
O6^i^—Zn1—O4	164.39 (12)	O5—C4—C3	114.9 (3)
O8—Zn1—O4	86.23 (12)	C6—C5—C1	115.6 (3)
O2—Zn1—O4	86.21 (11)	C6—C5—H5A	108.4
O9—Zn1—O1	167.91 (11)	C1—C5—H5A	108.4
O6^i^—Zn1—O1	83.38 (11)	C6—C5—H5B	108.4
O8—Zn1—O1	95.54 (12)	C1—C5—H5B	108.4
O2—Zn1—O1	76.98 (11)	H5A—C5—H5B	107.5
O4—Zn1—O1	81.02 (11)	O7—C6—O6	124.9 (4)
C1—O1—Zn1	105.3 (2)	O7—C6—C5	118.7 (3)
C1—O1—H1O	106 (3)	O6—C6—C5	116.3 (3)
Zn1—O1—H1O	109 (3)	C15—N11—C11	120.6 (4)
C2—O2—Zn1	114.9 (2)	N11—C11—C12	121.5 (4)
C4—O4—Zn1	130.7 (3)	N11—C11—H11	119.3
C4—O5—H5	109.5	C12—C11—H11	119.3
C6—O6—Zn1^i^	126.1 (3)	C11—C12—C13	118.8 (4)
Zn1—O8—H8A	127 (3)	C11—C12—H12	120.6
Zn1—O8—H8B	121 (3)	C13—C12—H12	120.6
H8A—O8—H8B	101 (5)	C14—C13—C12	118.9 (4)
Zn1—O9—H9A	137 (3)	C14—C13—C16	118.6 (4)
Zn1—O9—H9B	117 (4)	C12—C13—C16	122.5 (4)
H9A—O9—H9B	106 (5)	C15—C14—C13	119.6 (4)
O1—C1—C3	106.3 (3)	C15—C14—H14	120.2
O1—C1—C5	110.4 (3)	C13—C14—H14	120.2
C3—C1—C5	107.3 (3)	N11—C15—C14	120.7 (4)
O1—C1—C2	110.7 (3)	N11—C15—H15	119.7
C3—C1—C2	112.1 (3)	C14—C15—H15	119.7
C5—C1—C2	110.0 (3)	C16^ii^—C16—C13	124.9 (5)
O3—C2—O2	124.3 (4)	C16^ii^—C16—H16	117.5
O3—C2—C1	117.7 (3)	C13—C16—H16	117.5
O2—C2—C1	118.0 (3)	H1WA—O1W—H1WB	108 (5)
C4—C3—C1	115.6 (3)		
			
O9—Zn1—O1—C1	2.9 (6)	O1—C1—C3—C4	−52.5 (5)
O6^i^—Zn1—O1—C1	125.2 (2)	C5—C1—C3—C4	−170.6 (3)
O8—Zn1—O1—C1	−140.8 (2)	C2—C1—C3—C4	68.5 (4)
O2—Zn1—O1—C1	32.6 (2)	Zn1—O4—C4—O5	−147.6 (3)
O4—Zn1—O1—C1	−55.5 (2)	Zn1—O4—C4—C3	33.9 (6)
O9—Zn1—O2—C2	148.1 (3)	C1—C3—C4—O4	−11.8 (6)
O6^i^—Zn1—O2—C2	−108.9 (3)	C1—C3—C4—O5	169.6 (4)
O4—Zn1—O2—C2	55.8 (3)	O1—C1—C5—C6	55.0 (5)
O1—Zn1—O2—C2	−25.9 (3)	C3—C1—C5—C6	170.4 (4)
O9—Zn1—O4—C4	−170.7 (4)	C2—C1—C5—C6	−67.4 (4)
O6^i^—Zn1—O4—C4	1.6 (7)	Zn1^i^—O6—C6—O7	5.5 (7)
O8—Zn1—O4—C4	95.3 (4)	Zn1^i^—O6—C6—C5	−171.6 (3)
O2—Zn1—O4—C4	−78.3 (4)	C1—C5—C6—O7	152.2 (4)
O1—Zn1—O4—C4	−0.9 (3)	C1—C5—C6—O6	−30.6 (5)
Zn1—O1—C1—C3	86.9 (3)	C15—N11—C11—C12	0.5 (7)
Zn1—O1—C1—C5	−157.0 (2)	N11—C11—C12—C13	0.4 (7)
Zn1—O1—C1—C2	−35.0 (3)	C11—C12—C13—C14	−1.2 (7)
Zn1—O2—C2—O3	−164.3 (3)	C11—C12—C13—C16	176.8 (4)
Zn1—O2—C2—C1	13.4 (4)	C12—C13—C14—C15	1.2 (7)
O1—C1—C2—O3	−165.1 (3)	C16—C13—C14—C15	−176.9 (4)
C3—C1—C2—O3	76.4 (4)	C11—N11—C15—C14	−0.5 (7)
C5—C1—C2—O3	−42.9 (5)	C13—C14—C15—N11	−0.4 (7)
O1—C1—C2—O2	17.0 (5)	C14—C13—C16—C16^ii^	−173.2 (6)
C3—C1—C2—O2	−101.5 (4)	C12—C13—C16—C16^ii^	8.8 (9)
C5—C1—C2—O2	139.2 (3)		

Symmetry codes: (i) −*x*+1, −*y*+1, −*z*+1; (ii) −*x*, −*y*, −*z*+2.

### Hydrogen-bond geometry (Å, º)

**Table:**

*D*—H···*A*	*D*—H	H···*A*	*D*···*A*	*D*—H···*A*
O1—H1*O*···O6	0.93 (1)	1.80 (2)	2.626 (4)	146 (4)
O5—H5···N11^iii^	0.84	1.81	2.633 (5)	168
O8—H8*B*···O5^iii^	0.86 (1)	1.88 (1)	2.733 (5)	173 (5)
O8—H8*A*···O7^i^	0.86 (1)	2.58 (4)	3.031 (4)	114 (4)
O8—H8*A*···O7^iv^	0.86 (1)	2.04 (2)	2.866 (4)	161 (5)
O9—H9*B*···O1*W*^iv^	0.86 (1)	1.87 (1)	2.725 (5)	179 (5)
O9—H9*A*···O3^v^	0.86 (1)	2.57 (4)	3.145 (5)	125 (4)
O9—H9*A*···O2^v^	0.86 (1)	2.01 (1)	2.860 (5)	168 (5)
O1*W*—H1*WA*···O3	0.96 (1)	1.88 (1)	2.838 (5)	172 (5)
O1*W*—H1*WB*···O7^vi^	0.96 (1)	2.04 (3)	2.880 (5)	145 (4)
C14—H14···O9^i^	0.95	2.58	3.474 (5)	158
C12—H12···O3^vii^	0.95	2.52	3.390 (5)	152
C11—H11···O4^iii^	0.95	2.52	3.132 (5)	122

Symmetry codes: (i) −*x*+1, −*y*+1, −*z*+1; (iii) −*x*+1, −*y*+1, −*z*+2; (iv) *x*, *y*+1, *z*; (v) −*x*+2, −*y*+1, −*z*+1; (vi) −*x*+1, −*y*, −*z*+1; (vii) −*x*+1, −*y*, −*z*+2.
